# Analysis and Use of the Emotional Context with Wearable Devices for Games and Intelligent Assistants

**DOI:** 10.3390/s19112509

**Published:** 2019-05-31

**Authors:** Grzegorz J. Nalepa, Krzysztof Kutt, Barbara Giżycka, Paweł Jemioło, Szymon Bobek

**Affiliations:** 1 AGH University of Science and Technology, 30-059 Krakow, Poland; krzysztof.kutt@gmail.com (K.K.); bgizycka@agh.edu.pl (B.G.); pawel.jemiolo@gmail.com (P.J.); szymon.bobek@agh.edu.pl (S.B.); 2 Jagiellonian University, 31-007 Krakow, Poland

**Keywords:** affective computing, wearable sensors, affective gaming, context-aware systems

## Abstract

In this paper, we consider the use of wearable sensors for providing affect-based adaptation in Ambient Intelligence (AmI) systems. We begin with discussion of selected issues regarding the applications of affective computing techniques. We describe our experiments for affect change detection with a range of wearable devices, such as wristbands and the BITalino platform, and discuss an original software solution, which we developed for this purpose. Furthermore, as a test-bed application for our work, we selected computer games. We discuss the state-of-the-art in affect-based adaptation in games, described in terms of the so-called affective loop. We present our original proposal of a conceptual design framework for games, called the affective game design patterns. As a proof-of-concept realization of this approach, we discuss some original game prototypes, which we have developed, involving emotion-based control and adaptation. Finally, we comment on a software framework, that we have previously developed, for context-aware systems which uses human emotional contexts. This framework provides means for implementing adaptive systems using mobile devices with wearable sensors.

## 1. Introduction

Affective computing (AfC) is an interdisciplinary field, combining efforts in the areas of computer science, artificial intelligence (AI), and the social sciences (such as psychology). Originally proposed in 1997, in [1], affective computing is aimed at developing computational models of emotions [2]. Furthemore, using these models, emotional states can be recognized, synthesized, and interpreted [3]. Practical results in AfC can be particurlarly useful in a number of domains, such as human-computer interaction (HCI) and ambient intelligence systems (AmI), as well as serious and commercial video games, among others.

There has been a lot of interest and work in the area of AfC, due to the rapid progress of AI methods for classification, as well as the development of computer hardware for the multi-modal sensing of human conditions and activities. Of importance is the use of wearable sensors, together with new methods for data processing and interpreting, for mobile devices – which are constantly with the user. The purpose of this paper is to present our recent results in this area. Furthermore, we present some applications of these techniques in the area of game design. The motivation for the use of games is two-fold. First of all, games offer an immersive, yet controllable, environment for emotion elicitation, stimulation, and recognition. Secondly, they are an important area of application for AfC techniques, playing not only a role in commercial applications of video games, but also in the societal impact of so-called serious games for training and learning.

Despite the aforementioned points of importance, affective games still have not gained enough interest among players and significant developers [4]. A reason for this might be connected with the cost of the hardware that is necessary for such purposes. In a great number of experiments conducted in the affective gaming (AfG) field, laboratory-class equipment is needed due to its accuracy and possibility for multi-modal and simultaneous acquisition of various signals. For example, in [5], researchers used a ProComp Infiniti device to estimate player enjoyment preference, based on biological signals. In [6], the authors used a NeXus-10 to evaluate the experience of gaming in virtual reality. NeXus-10 was also used to implement games with an affective loop, see [7].

As the survey in [8] established, affective-adopting software, for players in the future, is expected to prove economically valid through the use of affordable and comfortable sensors, in order for them to be applicable in most recognizable AAA games (The term ‘AAA games’, read as ‘Triple-A’, coined in the late 1990s, refers to games developed with a large budget, dedicated to both production and promotion. Usually, such games feature high gameplay quality and sales). Visible progress towards commercial solutions has already been made. There have been some significant works that have tried to combine AfG mobile and wearable devices, which are characterized by unobtrusiveness. The authors of [9] used an Empatica E4 for detecting affective responses in patients with post traumatic stress disorder by a computer game. Another notable example could be Nevermind [10], a game that used sensors from wristbands and affordable depth cameras to introduce the affective loop. This was the first game on Valve’s Steam platform that incorporated methods of AfC, although, admittedly, the reviews were widely differing. It is also worth mentioning that a software for contact-less pulse measurement was designed by Poh, McDuff, and Picard in [11]; as heart activity is closely connected to the emotional state of individal, it is suggested that emphasis should be put on this method in the future.

Using wearable devices in AfC is a case that many researchers have become interested in recently, due to the many possible applications, such as ambient assisted living [12] or social analysis of a group of people [13], among other applications, in areas ranging from validation between such devices and medical-quality platforms [14,15,16], through emotion detection and recognition [12,14,17,18,19,20,21], to creating new apparatuses that enable unobtrusive data acquisition [16,22]; the last approach being an answer to the remarks presented in [8]. It is worth mentioning that, in terms of AfG, sensors incorporated into gloves and shoes or gaming equipment (such as the mouse or game pad), are much more appropriate for active gameplay.

Finally, personal assistants are emerging as an area of interest for AfC, especially regarding emotional and contextual information. For example, in [23], cognitive assistants designed for elderly people were proposed, which, considering the interpretation of a situation, improved their functioning (i.e., in terms of memory). There are also games which were designed with intent to prevent problems with cognitive abilities, see [24]. Liao et al. [25] suggested using music to reduce stress, which also could be implemented as a feature of a personal assistant.

Emotions, which are the foundation of AfC, are a topic that is difficult to tackle. To this day, psychologists, neurologists, and other researchers interested in affect cannot reach an agreement about the nature of emotions. Numerous prominent figures of science and philosophy have debated, for centuries, whether emotions are primarily physical [26,27], mental [28,29], or, perhaps, that they form a more complicated compound of these components [30,31]. In our research, we are relying on the assumption that emotions are both cognitive and physical. Consequently, we believe that emotions can be detected by observing physiological changes in the body, and can be interpreted within the specific context and information on where and when they occurred.

An important research problem is the detection of change in the emotional condition of a person, due to the fact that a range of different physiological signals can be the subject of analysis. Moreover, information fusion techniques should be used for their interpretation; in our case, we consider multi-modal detection, using information about heart activity and galvanic skin response. Furthermore, we are currently experimenting with the integration of information on the affective expressions of faces. To integrate and analyze this information, we use the context-aware systems paradigm [32,33,34].

We have to face several challenges, regarding the experiments that we perform: In order to discover the most significant data and signals that can provide cues for the affective states of a person, one needs a very controllable environment. For this purpose, we think that games (especially affective games) are an adequate solution, which enable both a high degree of control over the situation parameters and the natural feeling of the context, if designed carefully – allowing for natural affective interaction. Furthermore, with video games as a computer-supported research tool, we can use other devices to track the physiological activity of a person and compute it within the game engine.

In our work, we focus on the use of data regarding the affective condition of a person in two areas. The first area is the development of an affective loop in computer games, which improves the player experience. Furthermore, this mechanism can be used in serious games and training to improve the learning process. The second area is the use of this data in ambient intelligence applications, such as cognitive assistants. Incorporating information on the emotional condition of the user provides her/him with a more personalized experience. Moreover, it is crucial for gamers not to have their freedom of movement limited. The same applies to the users of cognitive assistants on mobile devices and other ambient intelligence applications. This is why, in our approach, we rely on the use of wearable sensors and devices.

This paper is partially based on preliminary results, previously reported in several conference papers that we reference in the subsequent sections. On the other hand, we introduce new results, including the concept of the game design pattern ontology, extended implementation and evaluation of affective loops using game prototypes with wearable sensors, details of the processing of affective data, application of the AfCAI (Affective Computing with Context Awareness for Ambient Intelligence) framework with emotional context in the AmI setting. Furthermore, we provide a full synthesis of the work that has been done by the authors in recent years, with more detailed results, evaluations, and conclusions which were not published before.

The rest of the paper is organized as follows: In Section 2, we discuss our main results regarding the analysis of emotional contexts. First, we discuss the methods and tools for affect detection in Section 2.1; then, we present our work in the area of computer games which use these methods in Section 2.2; including the prototypes of affective games we have developed in Section 2.3. We performed a number of experiments in this area, which are briefly described in Section 2.4. The affective data can also be used for context-based adaptation in AmI, as described in Section 3. We discuss our approach and results in Section 4.

## 2. Results in the Analysis of Emotional Context with Wearable Devices

In our work, we consider the use of wearable sensors for providing methods for affect detection and affect-based adaptation in AmI systems. We describe our experiments with a range of wearable devices, such as wristbands and the BITalino platform, for affect change detection, and discuss an original software solution which we have developed for this purpose. Furthermore, as a test-bed application for our work, we selected computer games. We present our original proposal of a conceptual design framework for games, the affective game design patterns. As a proof-of-concept realization of this approach, we discuss some original game prototypes, which we have developed, that involve emotion-based control and adaptation. Finally, we present a software framework, which we also previously developed, for context-aware systems that use human emotional contexts. This framework provides means for implementing adaptive systems by using mobile devices with wearable sensors.

### 2.1. Affect Detection Using Wearable Sensors

**Figure 1:**
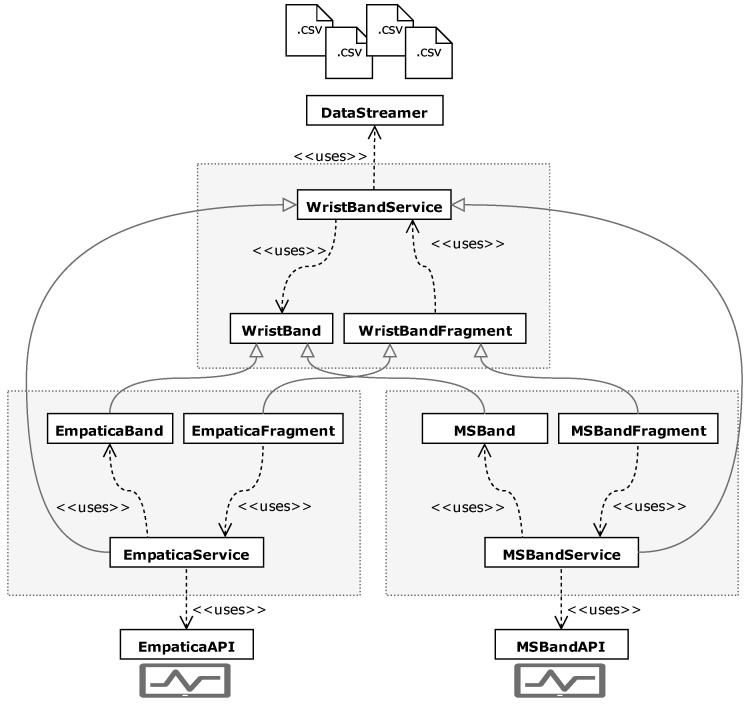
Modular architecture of BandReader [35].

With the growing interest in mobile technologies, one can observe a trend of increasing interest in using them in the laboratory context. On one hand, these devices are widely accessible and, therefore, can be used in real-life applications. However, their manufacturers often use lower-quality sensors to reduce the cost of such instruments. To address the need for comparing those devices and using them in studies, we created a software solution called BandReader, described in detail in [35] (see Figure 1), which enables us to collect data from wristbands and other non-medical platforms using Bluetooth technology and then broadcast the acquired statistics to all listening applications and devices.

Our previous preliminary research in field of AfC [36,37] led us to specify which software will be convenient for laboratory experiments. Among others, we established that such technology should facilitate recording data from several different tools simultaneously, take care of the fact that every wearable device provides another set of sensors, and save files in a convenient format (such as .CSV). Furthermore, the application should feature a modular architecture, so as to be easily expanded with new devices and allow synchronization with other data-sources. These assumptions are consequences of our earlier propositions regarding a mobile sensor platform – a modular design for affective data flow, described in detail in [38]. In such a stream, data is gathered by mobile sensors and then transferred to a processing unit which analyzes it and broadcasts it to be used by other platforms.

**Figure 2:**
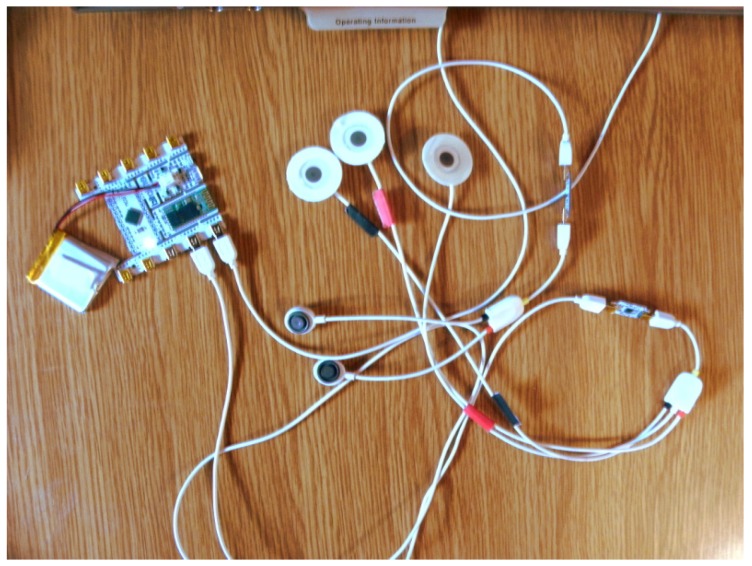
BITalino (r)evolution kit: Basic components [35].

**Figure 3:**
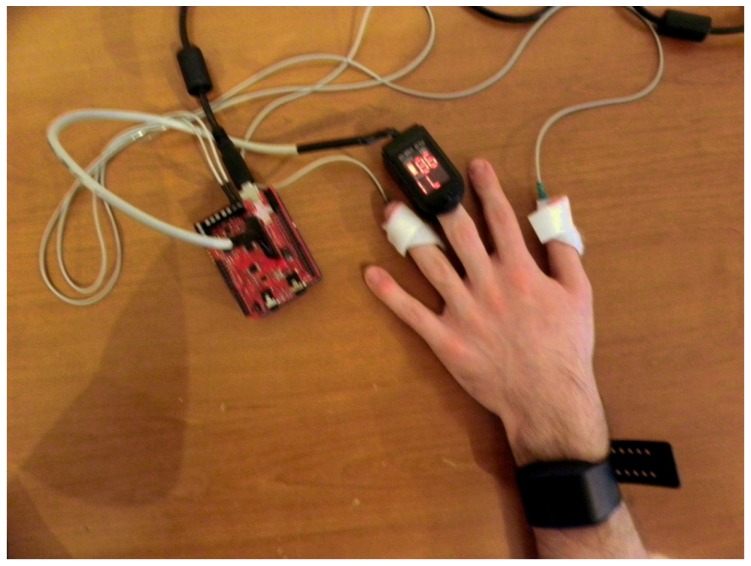
e-Health platform and Empatica E4 band, demonstration of devices equipped on the participant [35].

An important aspect of AfC systems is their ability to gather data, process it, and generate an answer dynamically, in real time, in a so-called affective loop. This method of human–computer interaction, understood in such a way, puts great emphasis on data collection, as the subsequent steps of interpretation and analysis depend on it. Therefore, we conducted a series of experiments enabling us to test the accuracy of several devices, such as the Microsoft Band 2, Empatica E4, e-Health, and the BITalino (r)evolution kit (see Figure 2 and Figure 3). It is worth emphasizing that the selected wearables offer real-time heart rate and galvanic skin response monitoring, as opposed to some fitness trackers, which offer only highly filtered and averaged data.

During a preliminary experiment, we tested 21 people by presenting them pictures from the Nencki Affective Picture System [39] and getting them to play London Bridge, an affective game created by us (see Section 2.2). As the results suggest (based on 7 out 21 test sessions), the BITalino (r)evolution kit remains to be the most prospective platform for measuring heart rate and galvanic skin response signals, compared to the Microsoft Band 2, Empatica E4, and e-Health platforms. In the same study (18 test sessions analyzed), we managed to establish that BandReader proved to be a reliable solution for AfC experiments, as it met our expectations as described above (i.e., sustaining a connection with used devices during whole session, multi-band readings, and generating proper data files). BandReader was also used in two previous phases of our experiments, with 6 and 9 subjects, respectively. However, we did not use simultaneous data acquisition from several devices in those experiments.

To summarize our contribution, our solution can simplify the implementation of practical AfC software on mobile devices, whilst also taking the affective aspects of interaction into consideration. It is a practical framework, which integrates multi-modal sensing from wristbands and wearables with mobile device processing. The modular architecture of BandReader allows the addition of more interesting devices to be tested and used in the future (i.e., board platforms like the BITalino (r)evolution kit, which we managed to establish as a possible solution for our future experiments, in combination with chest straps).

### 2.2. Designing Games with an Affective Feedback Loop

In order to evaluate the ideas related to the detection of emotions using mobile sensors, we decided to take up the area of computer games. Our approach is based on an earlier study, described in [37], which was our first attempt at using affective computing techniques in the design of video games. This was also the time when our original idea of the proposed concept for facilitating games with an affective component first sparked. We realized that video games provide a perfect experimental ground, both for validating initial ideas on affective game design and testing new measuring devices for emotion detection.

**Figure 4:**
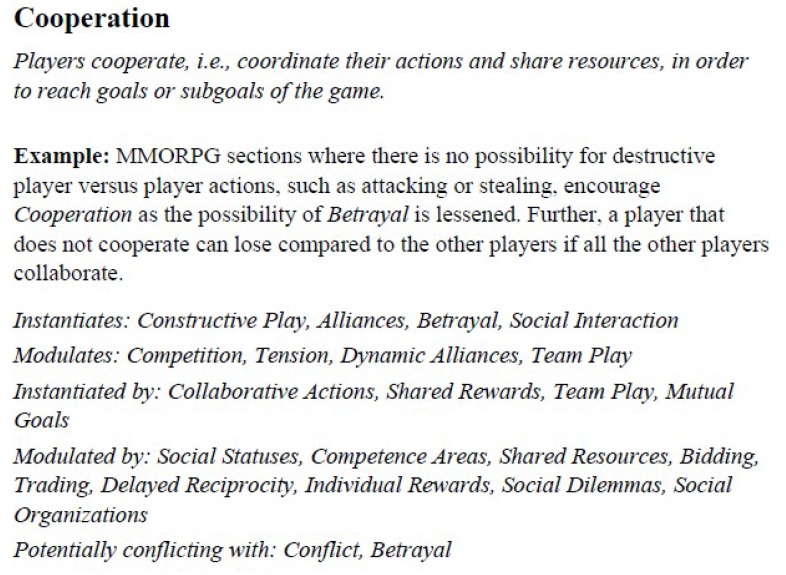
A template of a game design pattern.

**Figure 5:**
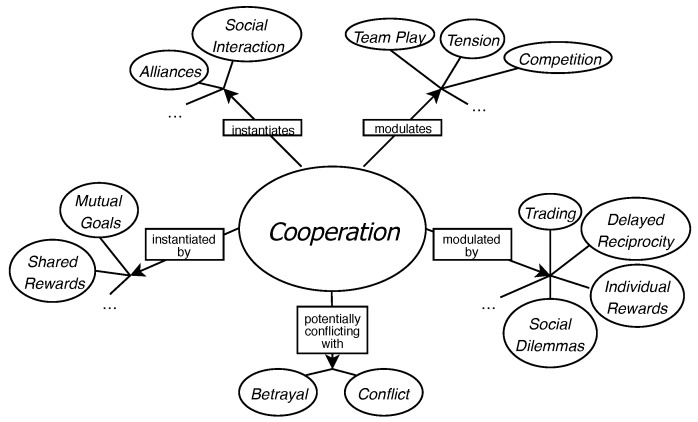
A diagram of the *Co-operation* pattern and its relationships with other patterns. Only selected patterns are depicted, those that do not appear in the figure are indicated by ellipses.

As a design framework for the experimental game, we chose the game design patterns, introduced by Björk and Holopainen [40]. By game design patterns, one should understand the mechanics and solutions to gameplay problems, which re-occur in various games and across different genres. These patterns form complex inter-relationships and hierarchies, which enables interesting and cohesive game design. For example, a pattern called *Cooperation* describes games in which the game goal can be achieved only by player collaboration. By using this pattern to engage the players in jolly cooperation, one also encourages them to form *Alliances* in order to achieve *Mutual Goals* and, at the same time, disfavors *Conflicts* between the players. Each of emphasized phrases refer to other patterns, which can instantiate, modulate, or conflict with each other. An example of a game design pattern template from the collection of Björk’s and Holopainen is presented in Figure 4 and Figure 5.

We suggest that certain patterns, by their nature, evoke emotional responses in the player, which we can detect using the proposed framework (Section 2.1). Using some of the identified affective game design patterns, we designed a scroll-runner game to verify our approach. In the game, several elements (means of representation of the player score, enemies, notifications of half-time dedicated for completing the level having passed, and so on) correspond to some of the patterns (*Indirect Information*, *Enemies*, and *Alarms*, respectively). We believe that each of these evokes an emotional response; in the listed cases, the level of stress of the player is raised. We decided to assume that the stress level can be interpreted in terms of basic physiological signals, as observed with our measurement devices. In our studies to date, we have collected galvanic skin response (GSR) and heart rate (HR) data from more than one hundred experimental participants, together with time-stamps of game events that correlated to certain affective game design patterns.

While we developed our account, we realized that an affective model of the player and affective model of the game-world, as well as of the game play, should be considered early in the design phase (in line with [41,42]). We propose that affective design patterns may serve as a framework for affective game design, enabling designers to tailor together those affective models. Building on our previous results and observations, we developed two prototype games, where we attempted to enrich the design with affective loops. By an affective loop, we understand a mechanism that enables natural and dynamic interaction between the player and the game-world, which react to the affective responses of each other. For example, if a high level of stress is detected in the player, this might result in the subtraction of some strongly-arousing game-world components (or part thereof), if the intention of the designer is to maintain player arousal within desired levels (for more on the role of intention of the designer in affective games, see [43]). Moreover, we implemented some physiological signal readings (muscle activity) into the game mechanics, in order to pull the player further into the affective loop.

**Figure 6:**
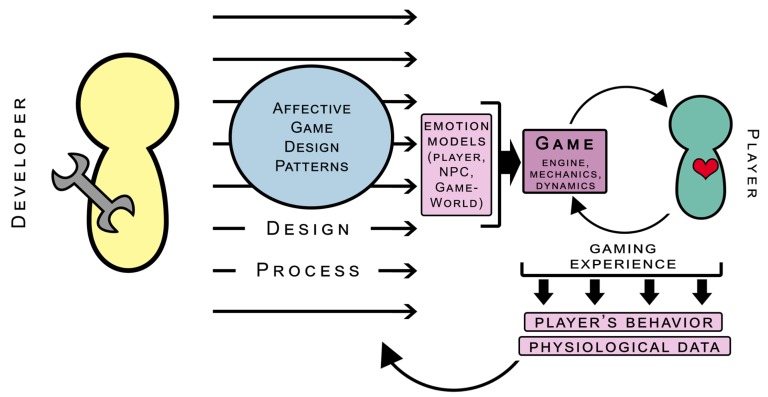
Affective game design patterns: Scheme of the concept.

We think of affective game design patterns as a design tool for game developers, which enables them to tailor emotional models into game design and gameplay, early in the design phase. The emotional models can refer to the player affect model, the NPC (Non-Player Character) affect model, or the game-world affect model, in general. The player affect model needs to accurately mirror the emotional state of the player, for which proper emotion detection methods are necessary. The NPC and game-world affect models both need to communicate with the player affect model, in order to affectively adapt themselves. Therefore, the NPC affect model has to be able to generate some responses, based on the interpretation of affective data coming from the player model. These responses may include both some visual NPC avatar changes (such as face expressions, behaviors, prosody, and so on), but also changes in the internal beliefs, attitudes, and so on, of the NPC. At the same time, the game-world affect model also receives information on the affective states of the player and the NPC, so that it can modify world parameters as well as other game components. The characteristics of these parameters will differ, depending on the specific game where the affective loop is realized. These may be weather conditions, field-of-vision effects (especially in FPS shooters) [44], enemy behavior [45], and so on; as the player reacts affectively to these changes, the player affect model receives new data, which is then forwarded to other affect models in the game. This is essentially how the affective game loop keeps on running. On a more general level, the behavioral data and physiological data of the affective responses of the player are constantly collected, which can provide information on the execution of affective game design patterns and improve them. For a graphical representation of the affective game design process influenced by patterns, see Figure 6.

**Figure 7:**
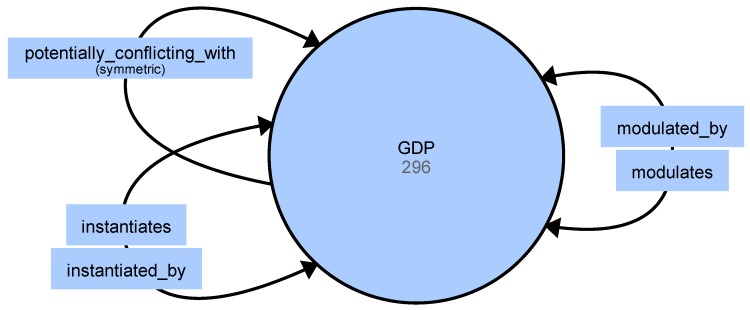
Game Design Pattern ontology scheme.

As such, we have our sights set on creating a computer-supported game design tool. For this purpose, we used Björk’s and Holopainen’s concept of game design patterns, and aimed to create an ontology of these patterns. This concept is understood in computer science [46] as a type of formalized knowledge representation, which enables reflecting of various relationships and hierarchies between concepts. This suits the nature of game design patterns, which form a complex web of inter-relations: some patterns instantiate others, or inhibit another from appearing, among other dynamics. In our case, a scope of the formal representation of the game design pattern collection is provided. On the technical level, it consists of one class (GDP, game design pattern), five relations (see Figure 7), and 296 individuals (specific patterns). Our ontology is prepared in OWL 2 (Web Ontology Language) [47], and is serialized using OWL/XML and Turtle notations, which are popular and supported by various frameworks and libraries. Such a model can be queried using, for example, the SPARQL syntax [48].

The benefit of using the game design pattern ontology is its potential for building interesting queries, in order to reflect the intentions of the designer. For example, suppose that the designer is planning a game that is based on *Traversing* the game-world, while *Collecting* some *Pick-Ups* along the way. Using a properly-constructed query to the ontology, one can gain knowledge that, in using such patterns, the designer could also use, for example, *Resource Locations* or *Supporting Goals*, since these are patterns that can be instantiated by *Pick-Ups*.

### 2.3. Affective Loop in Practice

An important step in our research was the developement of games that implemented an affective loop, which is described in full in [49]. As [50] indicated, emotions are usually accompanied by various reactions of the sympathetic and para-sympathetic systems, which became a point of reference for our work. One can observe those affective expressions on behavioral [51], cognitive [52], and physiological levels [26]. Based on that, we grounded our work in detecting changes in biological signals: muscle electrical activity, heart rate, and galvanic skin response. Combining these data with the interaction context (see Section 3) opens up the possibility to infer player emotions. This, in turn, allows for adjustment of the environment to the current state and closing the loop by affecting the user and re-detecting.

Our approach relies on rule-based systems for affective inference, drawing from explainable methods. Analogously to [7], we defined linguistic terms and a set of pre-defined rules to determine the emotional state of the user. Therefore, we refer to the dimensional model of emotions suggested by James A. Russell [53], which consists of two continua, called arousal and valence. The first relates to the intensity of emotion, while the second refers to it’s *coloring*; this means that, for example, anger consists of a low-pleasure component and a lot of intensity. Following [54], we assumed that changes in GSR and HR are connected to the arousal dimension, whereas valence corresponds to visual aspects and storyline, which can be defined as context [55]. In order to answer the contemporary need for adaptive, personalized experiences, we propose a solution using physiological signals measured with the BITalino (r)evolution kit, a non-medical device.

We also address the need for including individual differences, in terms of reactions to different stimuli. A calibration is performed to adjust the default parameter settings and set reference points for later readings. The baselines of the HR, GSR, and electromiography signals are calculated for some time before each game.

**Figure 8:**
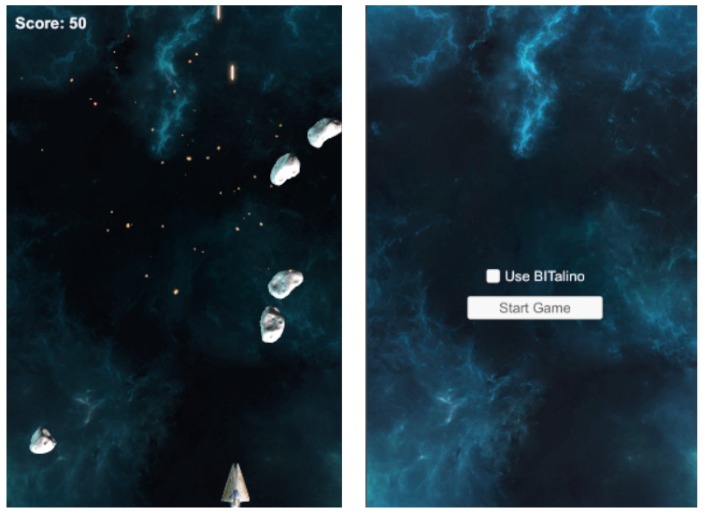
Space Shooter gameplay screenshot [49].

We prepared two game prototypes. The first is Space Shooter, which is a modification of the Unity arcade shooter [56], which enables the user to steer a starship in space. The player receives points for destroying oncoming asteroids (see Figure 8). Clenching of the fist allows the user to shoot, while changes in his HR are used to modify the speed of the asteroids. It is also worth mentioning that the brightness of the background changes, according to the calculated emotional index [57]. Grounded in GSR and HR, it produces values related to negative and positive emotions. As for the affective loop, the user reacts to the game and, at the same time, tries to control it. Faster falling of asteroids induces stress. This, along with background brightness changes, suggests that the user should try to control their heart level. Meanwhile, the player knows that they can tighten their hand if they want to shoot.

**Figure 9:**
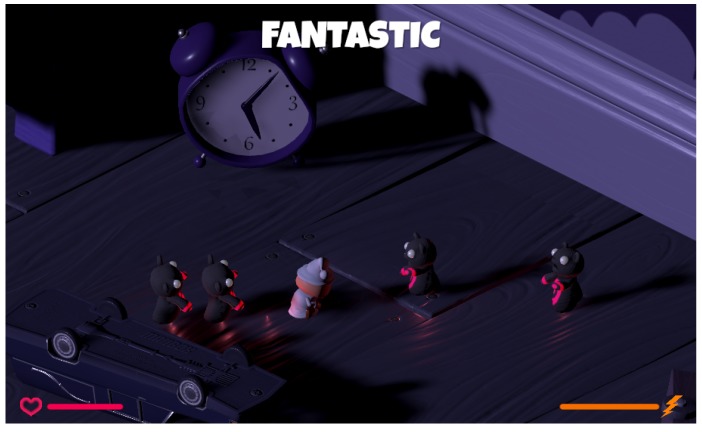
Freud Me Out gameplay screenshot [49].

Freud Me Out is another game prototype with a built-in affective loop, created by the authors (see Figure 9). It was developed using the basics of the Unity survival shooter guide [58]. The name of the game refers to a storyline derived from the psychoanalysis of Sigmund Freud. Throughout the game, the player is offered help provided by a psychotherapist, who clarifies the way the game-world works. For example, when the player is very frustrated or very angry, enemies slow down to adjust their speed to those emotions, and proper information is provided on the screen. It is a key point in the explainability of the affective loop implemented in our application, as well as in keeping the player interested. Changes in enemy movement affect the player, who relaxes and, therefore, their HR and GSR activity eases. Those current values are read and compared to the values calculated during the calibration phase. If they drop below a specified level, the mechanics related to the speed and randomness of the appearance of enemies are activated. Analogously to the previous prototype, the player is prompted to use a muscle-related mechanic, called SuperPower, which enables them to destroy a lot of opponents at once. In the non-affective version, SuperPower is enabled by a key-press.

A benchmark, in the form of a survey, was provided to verify our approach. Nine people (students, working young adults, and high school students) played the affective and non-affective versions of Freud Me Out and, then, completed a form divided into three dimensions: mechanic-type preference (two items), immersion (four items), and game level adjustment (four items). The results (see Table 1) suggested that the affective loop version of the game turned out to adapt better to current emotional state of player and was indicated to be more immersive than the prototype without the loop mechanism. Basing on the game logs, it occurred that the affective version of the mechanic were used 1.5 times more often than the non-affective equivalent (as inferred from the frequency of SuperPower usage). 

We presented an architecture for game prototypes with a built-in affective loop mechanism, based on biological signal detection. Our contribution also lies in introducing a calibration phase to account for individual differences and in extending the explainability of the integrated methods, thanks to a tutorial phase. Furthermore, we suggest that certain metrics can help to evalute affective game prototypes.

### 2.4. Experiment Summary

To date, we have conducted three series of experiments using our software and our experimental setup, continuously developing our protocol and improving the game prototypes. The experiments took place in April 2017, November 2017, and January 2018, as well as in March 2018, see Table 2. Currently (April 2019), another study is in progress.

#### 2.4.1. Pilot Study

In a preliminary study, we focused on testing our hardware of choice: Wearable wristbands (Empatica E4 and Microsoft Band 2) and our custom application (BandReader). The procedure consisted of a single phase, where participants evaluated their arousal, on a scale from 1 to 7 (where ’4’ referred to a neutral state, ’1’ to deep relaxation, and ’7’ to high arousal), in reaction to photographs displayed on a computer screen. The presented pictures were selected from the affective pictures database [39]. In further studies, this task was used as a reference (see Section 2.4.3). The visual stimuli presentation and evaluation scale were prepared using the PsychoPy (https://www.psychopy.org/) program. We examined six participants in this study, and collected GSR and HR/blood volume pulse (BVP) data, together with time-stamps of each photograph presentation.

#### 2.4.2. First Series

In the first attempt, we aimed to find an optimal hardware setup and sensor selection. For this, we first selected the Empatica E4 and Microsoft Band 2 wristbands, paired by Bluetooth to the BandReader application. Three phases contributed to the experimental procedure. The first phase, intended as a reference phase for further data analysis, was performed analogously to the picture evaluation from the pilot study. In the second phase, calibration was performed while basic signals where collected and user was asked to relax. In the last phase, participants played a simple scroll-runner game developed for research purposes (see Section 5). As in the case of the pilot study, we gathered GSR and HR/BVP data, as well as timestamps of visual stimuli appearance and of in-game events. The results from the first series and from the pilot study were published and discussed in [37]; these allowed us to configure our initial hardware setup and test the BandReader application.

#### 2.4.3. Second Series

In the second series of experiments, our goal was to include multi-modal sensory platforms (BITalino (r)evolution kit and e-Health) in the procedure. We compared their performances and validated the data quality against a medical-class device (Neurobit) and a wearable chest strap (Polar Pulse). The platforms provided data on HR/BVP and GSR, same as the wristbands, and the data was recorded on the computer on which the participants performed experimental tasks. We added new stage to the procedure, where participant was asked to look at a neutral picture from the Nencki Affective Picture System (NAPS) database and, after a specified amount of time, a sudden, unpleasant sound was played from the International Affective Digital Sounds (IADS) database [59]. The wristbands used in the previous study were included as well. Again, we used BandReader to acquire data. A total of 21 subjects took part in the experiment. Our observations on the experimental setup and quality of data provided by the used devices allowed us to choose BITalino as the best candidate for future research.

#### 2.4.4. Third Series

In this series, we aimed primarily for affective data acquisition, using our experimental setup of choice, consisting of BITalino, e-Health, and Empatica E4 (we decided to exclude Microsoft Band 2, due to its relatively poor data quality). Again, we collected HR/BVP and GSR data, as well as timestamps of four experiment phases: reference (the affective picture evaluation), calibration (baseline signals), the main phase (side-scroller game), and final phase (sudden, unpleasant sound). We examined 102 participants. The results of this series convinced us to focus on the use of BITalino, and refrain from using Empatica E4 (due to configuration problems) and e-Health (due to the lack of support). However, this series allowed us to consolidate the experimental protocol and identify proper affective data sources; that is, those suitable for processing in the context-aware framework.

#### 2.4.5. Fourth Series

In our last series of experiments, we tested one of our affective game prototypes, Freud Me Out. Participants performed calibration, then played, successively, the affective and non-affective versions of the game, following which, they filled in the form. We used BITalino to acquire the HR and GSR data. Additionally, we also recorded in-game logs with timestamps and surveys completed by participants. We examined nine people. We managed to establish that the game implementing the affective loop was perceived as more immersive and better adjusting to player abilities than the regular, pre-designed version. The full results from this series were published and discussed in [49]. This last series allowed us to gather user feedback, which will be helpful in the design of the final versions of the affective game prototypes that we are using currently to gather larger amounts of affective data. Furthemore, we are putting this data into the context of the personality of the player.

### 2.5. Sensory Data Processing

Emotion determination based on physiological signals requires proper data processing. Besides regular tasks, such as signal normalization and filtering, the two most important steps are feature extraction and prediction model preparation. Our analysis, performed in the time domain, resulted in a set of features that were most useful for predicting emotions.

In the ECG signal, we determined the interval between each two consecutive heartbeats (IBI, inter-beat interval), as well as the number of beats per minute (BPM) were most useful. Based on these, five features useful for emotion recognition can be calculated:

- the median of absolute deviation from the average IBI (MAD);
- the standard deviation of intervals between consecutive beats (SDNN);
- the root mean square of successive differences between consecutive R–R intervals (RMSSD);
- the standard deviation of successive differences between consecutive R–R intervals (SDSD); and
- proportion of differences between R–R intervals greater than 20 ms (pNN20) and 50 ms (pNN50).

Our GSR signal analysis begins with the tonal component removal. These are slow changes (from tens of seconds to minutes) – associated with, for example, skin hydration – which are irrelevant in the analysis of emotional states. What remains is the phasic component, that is rapid changes related to events (e.g., stimuli presentation). We can, then, identify the peaks in the signal and, as a result, calculate the following features:

- the number of peaks,
- the amplitude of the peak,
- the duration,
- the rising time,
- the falling time, and
- the delay between the occurrence of the stimulus and the appearance of the peak.

**Figure 10:**
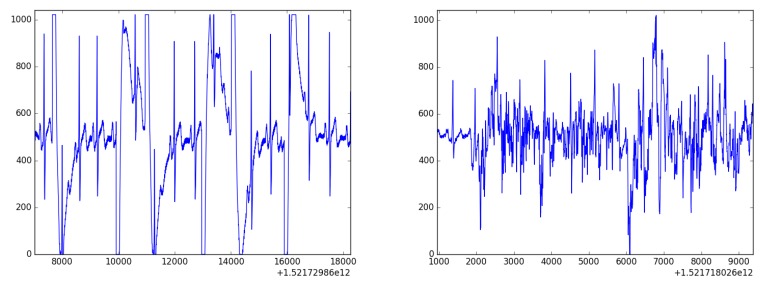
Two samples of ECG signal. R peaks are visible, but are surrounded by a large amount of noise.

**Figure 11:**
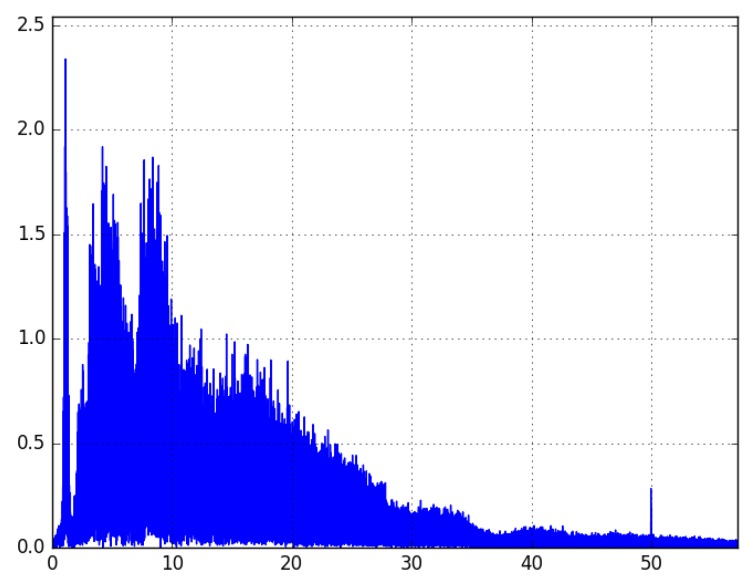
Noised results of the Fourier transform of a signal collected from one user.

All these features were used as an input for various machine learning models. We have tried, i.a., Decision Trees, Random Forest, Gradient Boosting, and AdaBoost. The prediction was made for nine classes; for three levels of arousal and three valence levels (low, medium, and high). The best performance was achieved by Gradient Boosting, leading to 34% accuracy in emotion prediction. This result was much better than baseline ZeroR (11%), but was still uncertain, due to the fact that we used cheap mobile devices (leading to unreliable skin contact or noise in the signal, see Figure 10 and Figure 11). To overcome this issue, we can combine various uncertain data to make the predictions stronger. This is achieved using Context-Aware Systems, as described in the following section.

## 3. Interpretation of Emotions Using Context-Aware Systems

As a more general solution for incorporating emotions into mobile systems, we proposed a platform called AfCAI (Affective Computing with Context Awareness for Ambient Intelligence), described in detail in [34], based on the notion of context-aware systems (CAS). These deal with the context (i.e.,“any information that can be used to characterize the situation of an entity” [60]), as stated by the most general definition. As in most of the modern state-of-the-art solutions, we aimed to deploy CAS on mobile systems. This facilitates the use of various types of context: (a) physical: measured by sensors, such as acceleration, GPS coordinates, and ambient light luminance; (b) environmental: obtained from relevant services, like current weather and road traffic; and (c) others: those stored in the device, such as events in a calendar or current phone usage. The proposed architecture is easy to extend with further sources of context. This may be the result of using a device that has a greater number of sensors (e.g., newer smartphones), connecting external sensors (e.g., pairing through the Bluetooth interface), or using new data sources (e.g., new web services). These objective data samples form a low-level context. We can synthesize and interpret them, providing a higher-level context; for example, “the user is at home” or “the user rushes to the exam and is late”.

Usage of the context makes the platform “emotion-ready”, from the point of view of Prinz’s theory of emotions (this theory was previously introduced in Section 1). First of all, one can easily provide a low-level context that is related to emotions, which can consist of data of various types: physiological data (HR, GSR) collected by external sensors, facial pictures taken by the phone camera, or voice characteristics provided by the microphone. These all refer to the form of emotions, or physiological changes that are observed by the agent. The second part of emotions in Prinz’s theory is the content of emotions; that is, the relationship between the agent and the environment. This relationship can be easily characterized, using other context data already available in the framework. As previously mentioned, based on this low-level context, a higher-level context can be generated; for example, “the user is at home and is relaxed” or “the user rushes to the exam, is late, and is stressed”.

**Figure 12:**
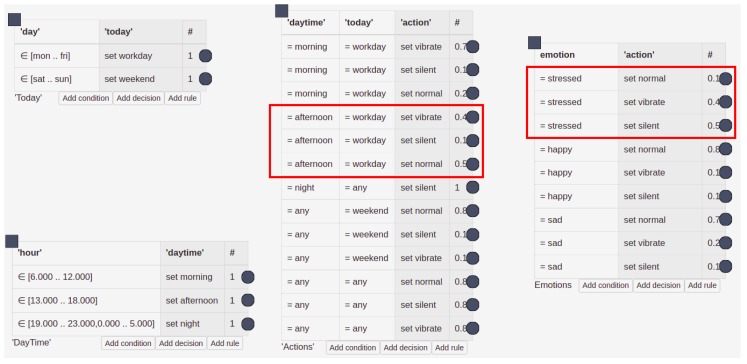
Simple context-aware model encoded in the HMR+ language. The column denoted with # contains the certainty factors of rules. The two red boxes represent rules that form a conflict set.

The collection of emotional data fits well into the area of CAS systems, which aims to provide the most accurate description of the current state of the system user, and further use it to modify the operation of such a system. With this in mind, attention must be drawn to the problem of data uncertainty: the most important aspects here being the measurement and interpretation uncertainties (for a discussion on uncertainty in CAS systems, see [61]). In the context of emotion, the first aspect is related primarily to the platform targeting on mobile devices. In this scenario, the sensors that are used are affordable for everyone (wristbands, BITalino), at the expense of resolution and measurement accuracy (see Section 2.5). The second one is related to inference based on uncertain data. The first problem is difficult to address at the software level. However, the second one can be solved by proper computational methods. Machine learning or fuzzy logic approaches can be considered, although these provide a kind of black-box solution, whereas what we aim at is a system that is transparent for the end-user. Our work in [62] indicated the HMR+ rule-based language (HMR+ stands for HeKatE Meta Representation. It is a textual representation of XTT2 models (eXtended Tabular Trees), a rule-based knowledge representation developed as a prior work to the research presented here. A sample model of this knowledge representation is depicted in Figure 12), which we have developed, is an optimal solution; it supports certainty factors and probabilistic interpretation possibilities, which provides a system that is well-balanced between handling uncertainty and explaining possibilities.

The proposed platform is a generic solution, easily adaptable to a specific setting. In particular, thanks to the use of probabilistic models, the sources of contextual data can be treated as separate blocks, which can be easily exchanged. This will not affect the whole model, only the resulting accuracy of the emotion prediction. Therefore, we see a wide range of possibilities of using the platform in AmI. In general, the basic applications for the AfCAI platform are all human-centric interfaces, which should make them more immersive. We see that it can be used in various types of intelligent assistants, who will respond better to user behavior and adapt to it. In our works, we have used the platform as a backend for emotional processing in various applications: computer games (see Section 2.3), experimental protocols related to the field of psychology [38], a music player with mood-based recommendations [63], and a visualizer of affective data on a spatial map, indicating which places in the area are related to what kind of emotions [64].

We have demonstrated that it is straightforward to include emotional contexts in AmI systems built on top of HMR+ and HeaRTDroid [65], mainly due to the modularized nature of HMR+ models, which can be easily extended with additional knowledge. Figure 12 presents a simple HMR+ model for adapting a mobile phone ringtone to a temporal context; this example came from the tutorial for HeaRTDroid, and originally did not include the emotional context table. As shown in the Figure 12, including additional components to the model does not (or hardly) affect the original model.

The model switches the mobile phone ring-tone mode to be either silent, vibrate, or loud, depending on the hour and day of the week. The original model contained three tables: DayTime, Today, and Actions. These tables contain rules that are executed to produce desired action for switching the ring-tone mode. For instance, at 3 P.M. on Monday, the first rule from the table DayTime will be triggered, then the first rule from table Today and, finally, the sixth rule from the Actions table, being the one with the highest certainty factor. This reasoning chain will switch the mode to silent.

The model presented in Figure 12 contains an additional table, called emotions. This table modifies the ring-tone, depending on the emotions of the user, and can be added without modification of the original model. It is executed independently of the other tables, generating ambiguous results that are resolved with HeaRTDroid and certainty-factor algebra [66].

For example, in the case discussed above, but with the additional information about the user being stressed, a conflict set will be discovered in the reasoning process, denoted by the red boxes in the Figure 12. The conflict set resolution is performed according to certainty-factor algebra and, as a result, the mode vibrate will be chosen, being the one having the largest certainty factor (0.64). Such an approach allows the seamless embedding of emotional context into the system, as the reasoning mechanism takes care of the appropriate ambiguity resolution.

**Figure 13:**
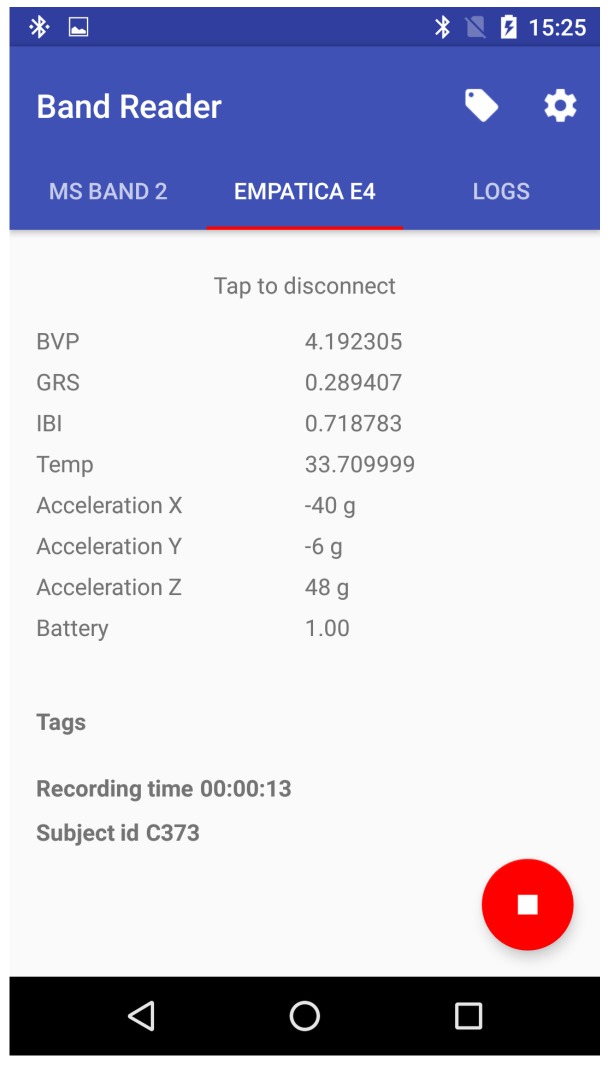
BandReader application online: physiological signals from Empatica E4 are being collected [35].

**Figure 14:**
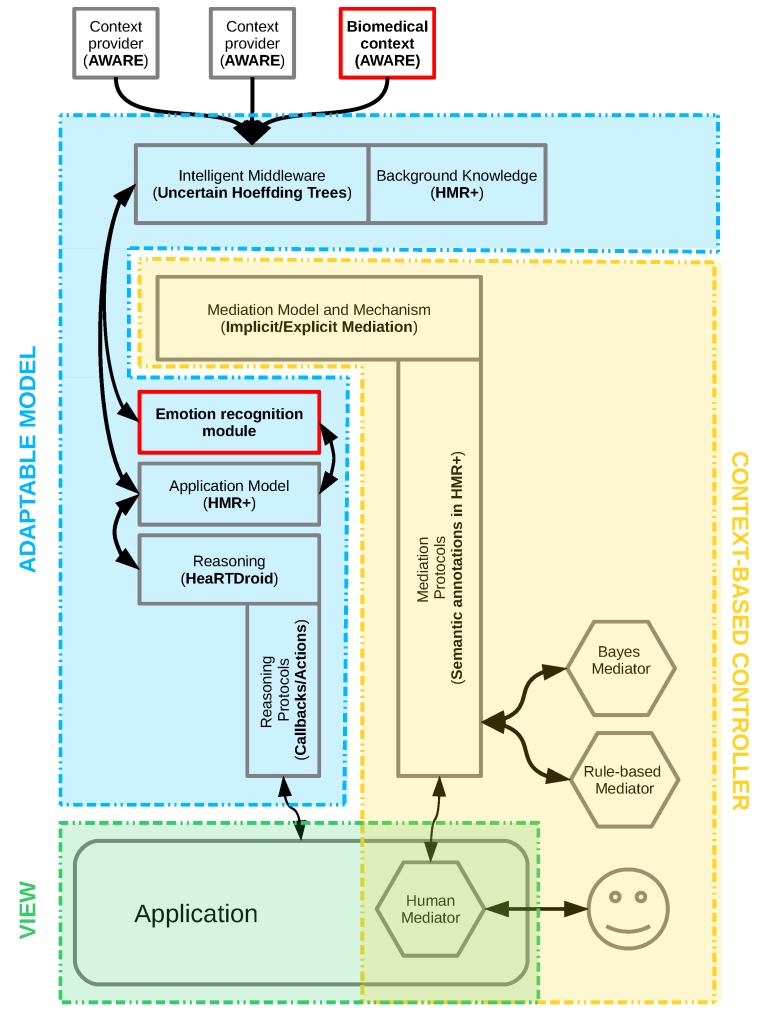
AfCAI (Affective Computing with Context Awareness for Ambient Intelligence) platform architecture [34].

Our proposed architecture is based on low-level context data, collected by various sensors or sub-systems of a mobile device. In our proof-of-concept solutions, they are collected by, among others, the BandReader application developed for gathering physiological context data from wearable sensors (see Figure 13). All signals are then consolidated, using an opensource AWARE framework (http://www.awareframework.com). Finally, the inference is carried out using the HeaRTDroid rule engine with the HMR+ language for expressing rules. The whole architecture is depicted in Figure 14 (for technical details of all mentioned software, see Section 5).

## 4. Discussion of the Approach

Our work is based on the hypothesis that the combination of technologies developed in the fields of AfC and context-aware systems will allow the introduction of new affective qualities into games, intelligent assistants, and other AmI systems.

The emotional dimension of contact with the user is important, but difficult to handle, in proper HCI design. Many studies in psychophysiology or AfC have tried to solve the problem of identifying user emotions. Most of them, however, were based on research conducted in a very controlled environment, where the subject was connected to a professional equipment and had limited movement. To make the system more accessible, the use of cheap mobile devices has been proposed, and the best solution would be to use technology that does not bother the user at all. The use of smartwatches and smartbands is promising. However, as our results indicate, this is more of a plan for the future [38]. It is currently an unstable market, where long-term support for devices is not guaranteed in any way. In addition, the data are of poor quality. Currently, a reasonable compromise between convenience, price, signal quality, and stability of support are open platforms, such as BITalino.

Due to the rapidly changing market and the number of available solutions, it is important to prepare an appropriate architecture. It should be adequately flexible and resistant to changes in hardware configurations, as well as stable enough to be a back-end for the final applications that will use information about emotions. We propose our AfCAI platform to be such an “emotion middleware”. It addresses several issues that arise in this area:

- By design, it is a mobile platform targeted for smartphones, so it can be used in everyday situations if the user has a proper device.
- Privacy is provided, primarily due to the processing of all data on a mobile device, without sending them over the network.
- BandReader and AWARE (see Section 5) are used for gathering data, which have a modular structure and are easily expandable to subsequent data sources by adding appropriate plugins.
- The handling of the uncertainty of measurement data (e.g., due to poor sensor contact, noise, or ambiguity of measurements) is one of the assumptions made.
- The prediction of emotions is not dependent on one data source, but on the entire collected context, which is processed with the use of inference by the HeaRTDroidrule engine.
- The use of an inference engine allows one to trace why the system behaved in such a way. It makes the system transparent for users and, as a result, their attitudes are more positive and they will be less cautious of this technology.

Based on the AfCAI platform, specific applications may be developed. As was indicated in Section 1, people are skeptical about intelligent assistants, because interactions with them are washed out of emotions and, as a consequence, they do not feel realistic. Even if the application monitors user emotions, it often does not use this information to modify its operation mode. To address this issue, we propose the use of an affective feedback loop. In this design, the user is not only watched, but also the whole environment may be changed to reflect its current state. These changes can be targeted so that the user feels the right emotions. Appropriate modification of behavior, however, is not trivial. In the case of computer games, which we have chosen to use to test the concept, game design patterns can be considered as such a protocol. They describe many possible building blocks, their inter-relations, and also the expected results (i.e., the user behavior). Our results indicated that people find games with an emotional loop more immersive and adjusting and, therefore, these games provide more fun for the players.

The use of the proposed technologies may lead to the improvement of the quality of life. Intelligent assistants are becoming available for many devices, starting from personal computers and smartphones, through to intelligent fridges to smart kettles. The use of knowledge about the user emotions can lead to less frustrating contact with technology on the one hand and, on the other hand, technology will probably help us to take care of ourselves and our mental state. After a hard day at work, an intelligent assistant in the car will guide us along the green meadows. Our smart home will dim the lights, adjust the temperature, and play smooth jazz, as it knows that this protocol (user pattern) will best suit the mood of the user.

As a more sophisticated use-case of an intelligent assistant, the proposed platform may form a basis for online therapists. Such an application could not only adapt to the emotions of the user, but also modify her/his affective state. This is a kind of affective loop, as was described above (in the case of games). However, in this case, the change of the state of the user would not be applied in the playing environment, but to an appropriately-prepared therapeutic protocol. Such a solution may be an interesting use-case for mental health prevention and as first aid for those who are afraid of real therapy, those who cannot afford it, or those who do not have close access to a psychotherapist. It will not replace real psychotherapy, but it will help people cope with overwhelming emotions.

In the games context, incorporation of the presented methods will lead to preparing more challenging (or more assisting) games, which will be both more immersive and better suited to the skill level of the user. These ideas are a manifestation of design-level heuristics, as presented in [43]. Incorporation of emotional detection and adaptation could become a remedy for derivative productions in times of great competitiveness; being a consequence of rapid growth in the entertainment industry.

Regarding our future research, we plan to explore other techniques and methods for acquiring affective data and developing our experimental game environment. Among others, we are interested in incorporating heart rate variability (HRV) analysis, which is indicated in the literature as a potentially useful characteristic [67]. HRV is a measure of variation regarding the time between individual heartbeats. It is a fairly simple indicator to compute. However, difficulties regarding its interpretation are persistent, as is the case for other physiological signals. HRV is most often associated with cognitive load or mental stress [68,69].

Another modality of our interest is breathing and respiratory patterns. Many studies have already incorporated these [70,71], which is probably thanks to their relative ease of use: respiratory sensors are often unobtrusive, as they take a form of, for example, a rubber band or a stretch belt. Deeper breathing is often associated with relaxation, whereas fast and shallow breathing might indicate high arousal, and so the level of anxiousness or anger can be inferred. Some studies report breathing (together with GSR) to be less reliable, in terms of accuracy, when detecting emotional stress, though [72].

We also intend to expand our studied modalities by face recognition, as facial expressions are one of most obvious observable phenomena that accompany emotions [73]. Research on face feature analysis (even in real time) has advanced significantly, using adapted machine learning methods as well [74,75,76,77]. Face recognition can be used not only for intelligent assistants, but also for more efficient affective communication (i.e., in interactive games and online games) [78]. Currently, we are experimenting with the use of services from Affectiva (http://affectiva.com) and MS Cognitive Services (https://azure.microsoft.com/en-us/services/cognitive-services/face).

We acknowledge that physiological and behavioral signals, alone, can be further facilitated by the deliberate reports of the subject, even if the reports may be biased [79]. Supplementary surveys or questionnaires might have a positive impact on the usability and efficiency of future affective systems [80]: using several modalities, together with psychological measures, seems to be a reliable means for capturing the specific traits, habits, behavioral patterns, and so on, of an individual. Each of these, we think, might influence everyday affective responses. Paying attention to the whole range of modalities available provides a great possibility for enhancing the predictive features of affective systems.

Moreover, we want to further build on the developed affective game design patterns and pattern ontology, so as to improve their usability in the design process. We see potential applications of our framework based on game design patterns in numerous fields, including serious games.

Finally, after the next phase of experiments, we hope to have enough multi-modal affective data to build basic classifiers of emotions. We are starting with the replication of findings in the literature [54,81,82,83,84,85,86,87,88,89,90], although the experimental setup differs with every experiment. As such, there is a need to build our own classifiers, aligned with our configuration.

## 5. Materials and Methods Used

In our works, we used the methods and tools described below. All of them, except the AWARE framework, were developed by the authors.

**BandReader** (https://geist.re/pub:software:bandreader) is a software solution for mobile devices that allows for data acquisition from selected wristbands. It uses Bluetooth technology to connect with supported devices and is able to gather data simultaneously from all of them. Thanks to its modular architecture, it can be easily expanded to co-operate with new apparatuses.

**Pattern Ontology** (https://www.affcai.eu/doku.php?id=sci:ontology) is a formal representation of the game design pattern collection, as proposed by Björk and Holopainen. Ontology has only one class: the GDP (Game Design Pattern), and all patterns are instances of it.

**London Bridge** (https://www.affcai.eu/doku.php?id=sci:londonbridgel) is our first game developed for research purposes, created using the Game Maker Studio software. It is a scroll-runner platformer game, designed with several affective game design patterns in mind, although it does not fully adopt affective loop assumptions.

**Affective game prototypes** (https://www.affcai.eu/doku.php?id=sci:prototypes) (namely, Space Shooter and Freud Me Out) are modified versions of Unity tutorial games, created for scientific purposes. They implement affective loops, in order to create an immersive experience for the player, by use of HR, GSR, and muscle-work signals.

**Basic experimental protocol** is a formal procedure created by us, to test our hypotheses. It consists of separate phases, namely a calibration stage, where the user is asked to relax and base signal values are calculated; then, the person being examined is presented with auditory and/or visual stimuli from standard databases; and, finally, they play a game.

**HeaRTDroid** (http://heartdroid.re) is a rule-based inference engine designed especially for mobile devices; however, it can also be run as a stand-alone desktop application. It uses a rule-language that can capture the dynamics of contextual information, uncertainty, and the incompleteness of data. The design phase is supported by the graphical web-tool.

**AWARE** (http://awareframework.com) is an Android framework that captures hardware-, software-, and human-based data, which is then analyzed using AWARE plugins.

## Figures and Tables

**Table 1:** Survey answers, according to separate dimensions [49].

	With Affective Loop	Without Affective Loop	No Difference
**Favorite mechanics**	17	1	n/a
**Immersion generation**	18	0	18
**Game level adjusting**	15	2	19

**Table 2:** Experiments series on Affective Computing. NAPS, Nencki Affective Picture System.

	Pilot Study	First Series	Second Series	Third Series	Fourth Series
**Number of participant**	6	9	21	102	9
**NAPS phase**	*✓*	*✓*	*✓*	*✓*	
**Calibration phase**		*✓*	*✓*	*✓*	*✓*
**Game phase**		*✓*	*✓*	*✓*	*✓*
**Sudden audio phase**			*✓*	*✓*	
**Bandreader**	*✓*	*✓*	*✓*	*✓*	
**Microsoft Band 2**	*✓*	*✓*	*✓*		
**Empatica E4**	*✓*	*✓*	*✓*	*✓*	
**BITalino (r)evolution kit**			*✓*	*✓*	*✓*
**e-Health platform**			*✓*	*✓*	
**London Bridge**		*✓*	*✓*	*✓*	
**Freud Me Out**					*✓*

## Abbreviations

The following abbreviations are used in this manuscript:

AI	Artificial Intelligence
AfC	Affective Computing
AfCAI	Affective Computing with Context Awareness for Ambient Intelligence
AfG	Affective Gaming
AmI	Ambient Intelligence
CAS	Context-Aware Systems
GDP	Game Design Pattern
GSR	Galvanic Skin Response
HMR+	HeKatE Meta Representation
HR	Heart Rate
HRV	Heart Rate Variability
NPC	Non-Player Character
